# A 5-week Non-Surgical Approach towards Denture Induced Hyperplasia

**DOI:** 10.2174/1874210601711010151

**Published:** 2017-03-31

**Authors:** Carolina Mayumi Iegami, Regina Tamaki, Pedro Tortamano Neto

**Affiliations:** Department of Prosthodontics, School of Dentistry, University of Sao Paulo, Sao Paulo, SP. Brazil

**Keywords:** Complete denture, Oral disease, Denture rebasing, Hyperplasia, Elderly, Vacuum chamber

## Abstract

**Background:**

Despite the standard approach towards denture-induced hyperplasia being surgery, as elderly population increases the systemic problems are carried along. Thus, surgery might be risky for patients with medical conditions.

**Objective:**

In this report, a patient with severe inflammatory papillary hyperplasia, medical problems and dental fear was treated with a 5-week nonsurgical protocol.

**Method:**

Once in a week, the upper denture was relined with a zinc enolic paste, for four weeks. In the fifth week, the denture was relined with fast set polymethyl methacrylate resin instead of zinc enolic paste so that the material would last longer than only a week until the new pair of dentures was manufactured.

**Results and Conclusion:**

The generated pressure combined with antioxidant and anti-inflammatory properties of the paste led to the elimination of the inflammatory papillary hyperplasia completely, satisfying the patient and allowing the manufacturing of a new set of complete dentures.

## INTRODUCTION

Old and ill-adapted dentures injure the mucosa and might cause denture related inflammatory papillary hyperplasia [1, 2]. The lesions are usually nodular and firm, with color varying from pink to red [1]. These lesions are usually induced by negative pressure between the tissue and ill-adapted denture or in vacuum chamber used in the upper denture [3]. Vacuum chambers consist in a palatal relief in the denture. At first, the chamber creates negative pressure in the area, enhancing retention [4]. However, with time, the negative pressure causes an inflammatory papillary hyperplasia, being painless and taking the form of its chamber [3]. It can also be associated with the colonization of *Candida albicans*, causing denture stomatitis and angular cheilitis [5]. Depending on the size of the lesion, removal of the denture or a simple relining might solve the problem although in extensive cases such as vacuum chambers related hyperplasia, the surgical approach is the standard treatment.

As elderly population increases, the systemic problems are carried along, thus surgery might be risky for patients with medical conditions. Cryotherapy and electrocauterization have also been suggested as treatment options for this condition [2, 6], but are almost as invasive procedures as surgery. This case report describes a 5-weeks non-surgical approach towards inflammatory papillary hyperplasia caused by vacuum / palatal chambers.

## CASE REPORT

A 70-years old male was enrolled to treatment for a new pair of dentures in the Department of Prosthodontics, School of Dentistry, University of Sao Paulo. The patient had worn his dentures for more than 20 years and as a result presented severe occlusal wear, reduced vertical dimension and ill adapted denture bases Fig. (**1**). During the clinical examination, it was noticed that the upper denture presented a vacuum chamber in the palatal area filled with an inflammatory hyperplasia (Figs. (**2a** and **b**). The patient reported having hypertension, fear of oral surgery and agreed to a nonsurgical approach since the surgical treatment was discarded.

Once a week, the upper denture was relined with a zinc oxide-eugenol paste (ZOE) (Pasta Lysanda, Lysanda Produtos Odontologicos Ltda, Brazil) for 4 weeks Fig. (**3**). In each session, the paste was removed and a new layer of paste was inserted. The patient was instructed on oral hygiene and to clean the denture carefully in order to not to remove the ZOE layer. In the fourth week, the lesion had reduced substantially Fig. (**4**); in the fifth week, the denture was relined with fast set polymethyl methacrylate resin (JET; Artigos Odontologicos Classico Ltda, Brazil); in the sixth week, the fabrication of the maxillary and mandibular complete dentures started. When the new set of dentures was inserted, the patient was instructed one more time on oral hygiene and denture maintenance.

This study was approved by the Ethics Committee of University of Sao Paulo, School of Dentistry (CAAE: 51323915.8.0000.0075).

## DISCUSSION

The elderly population is the main group of denture wearers and also, due to its advanced age, the systemic conditions are present. The surgical removal is the main treatment for denture related hyperplasia; however, it might not be ideal for individuals with systemic problems. Denture removal, cryotherapy and electrocauterization have also been suggested as treatment options for this condition [2, 6, 7]. When the alternative treatment was proposed, the patient preferred it for its less invasive approach and for being able to still use his denture. As a result, the patient became motivated and cooperative following instructions of oral hygiene and follow up sessions.

ZOE is usually used as an impression material constituted by zinc oxide and vegetable or mineral oil in one tube and eugenol and rosin in the other tube [8]. Other applications include use as a surgical dressing, bite registration, temporary filling material and temporary relining material for dentures [8]. The clinician is able to control setting time and consistency of the paste when needed. A paste of a thick consistency or high viscosity is able to compress the tissues, while a fluid material results in a detailed impression of the tissues [8].

Regarding other non-surgical approaches, the use of antifungal medication associated with denture removal was discarded because the patient was uncomfortable. Denture absence would affect his social interactions as well as oral functions. Also, it could take longer for the total regression of the lesion, due to its size. The use of tissue conditioners is a non-surgical alternative, however it was not an option in this case because the technique required pressure application, which is not given by the material. Tissue conditioners usually absorb impact caused by mastication [9]. An additional approach was suggested with heat-processed hard reline of the denture, showing the total regression of the lesion in 5 months, with visitations 2 to 3 weeks apart [10]. In the case report presented here, by changing the ZOE layer in the upper denture weekly, a constant and more dynamic positive pressure was created to stimulate a quicker atrophy of the inflammatory papillary hyperplasia Fig. (**4**). The zinc oxide-eugenol paste was chosen for being easier to be removed, whereas relining materials have more adherence to the denture base. Moreover, for being eugenol based, the paste presents antioxidant and anti-inflammatory properties, which benefit the treatment [11].

While this technique was applied for inflammatory papillary hyperplasia, regions it could be also applied to treat inflammatory fibrous hyperplasia, as the conditions are closely related [12].

In the fifth week, the denture was relined with fast set polymethyl methacrylate resin instead of ZOE so that the material would last longer than only a week until the new pair of dentures was manufactured. In order to avoid recurrence of the lesion, the new pair of dentures was made according to the technique used at the School of Dentistry, University of Sao Paulo. A preliminary impression was taken with an impression compound and the basal area was determined. A custom tray was made and the final impression was taken with a fluid impression material in order to copy every detail of the tissue. In order to not only promote retention but also to enhance stability, teeth were set in bilateral balanced occlusion. Furthermore, it is important to stress to the patient that post-insertion visits are required in order to make the proper adjustments of the dentures for a successful and lesion free outcome.

## CONCLUSION

The method provided a good result as an alternative non-surgical treatment for denture induced hyperplasia.

## Figures and Tables

**Fig. (1):**
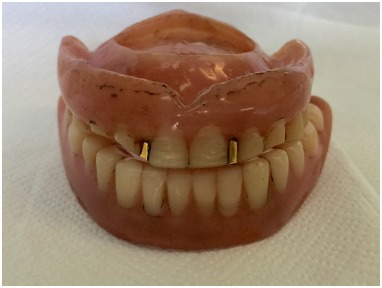
Patient’s old dentures.

**Fig. (2):**
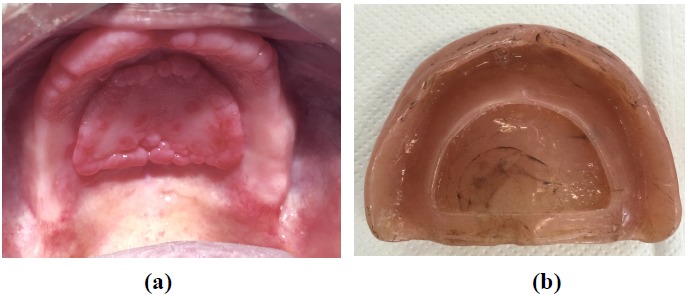
(a) Inflammatory papillary hyperplasia caused by vacuum chamber. (b) Upper denture with chamber.

**Fig.(3):**
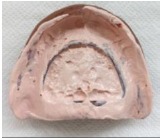
Relined denture.

**Fig.(4):**
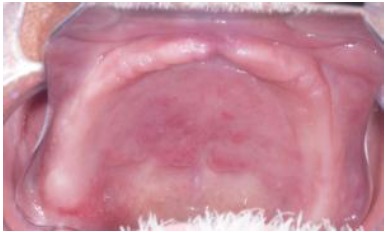
Aspect of the hyperplasia after five weeks.
